# Health care use and treatment-seeking for depression symptoms in rural India: an exploratory cross-sectional analysis

**DOI:** 10.1186/s12913-020-05162-0

**Published:** 2020-04-06

**Authors:** Tessa Roberts, Rahul Shidhaye, Vikram Patel, Sujit D. Rathod

**Affiliations:** 1 Health Services and Population Research Department, Institute of Psychiatry, Psychology & Neuroscience, King’s College London, De Crespigny Park, London, SE5 8AF UK; 2 Department of Research, Pravara Institute of Medical Sciences, Loni, Maharashtra India; 3 Department of Global Health and Social Medicine, Harvard Medical School, Boston, USA; 4 Department of Population Health, Epidemiology & Population Health Faculty, London School of Hygiene & Tropical Medicine, London, UK

**Keywords:** Depression, Help seeking, Health service utilisation, India

## Abstract

**Background:**

There is a large “treatment gap” for depression worldwide. This study aimed to better understand the treatment gap in rural India by describing health care use and treatment-seeking for depression.

**Methods:**

Data were analysed from a two round cross-sectional community survey conducted in rural Madhya Pradesh between May 2013 and December 2016. We examined the proportion of individuals who screened positive for depression (≥10) on the Patient Health Questionnaire (PHQ-9) who sought treatment in different sectors, for depression symptoms and for any reason, and compared the latter with health service use by screen-negative individuals. We analysed the frequency with which barriers to healthcare utilisation were reported by screen-positive adults. We also analysed the association between seeking treatment for depression and various predisposing, enabling and need factors using univariable regression.

**Results:**

86% of screen-positive adults reported seeking no depression treatment. However, 66% had used health services for any reason in the past 3 months, compared to 46% of screen-negative individuals (*p* < 0.0001). Private providers were most frequently consulted by screen-positive adults (32%), while only 19% consulted traditional providers. Structural barriers to healthcare use such as cost and distance to services were frequently reported (54 and 52%, respectively) but were not associated with treatment-seeking for depression. The following factors were found to be positively associated with treatment-seeking for depression: higher symptom severity; lack of energy, lack of interest/pleasure, low self-esteem, or slow movements/restlessness on more than 7 days in the past 2 weeks; being married; having discussed depression symptoms; and reporting problems with medication availability and supply as a barrier to healthcare. No evidence was found for an association between treatment-seeking for depression and most socio-economic, demographic or attitudinal factors.

**Conclusions:**

These findings suggest that the majority of adults who screen positive for depression seek healthcare, although not primarily for depression symptoms, indicating the need to improve detection of depression during consultations about other complaints. Private providers may need to be considered in programmes to improve depression treatment in this setting. Further research should test the hypotheses generated in this descriptive study, such as the potential role of marriage in facilitating treatment-seeking.

## Background

Depressive disorders are largely untreated despite accounting for an enormous burden of disease. The 2010 Global Burden of Disease study found that depression was the second leading cause of disability worldwide [1]. However, in developed countries only 54.3% of people with a 12 month major depressive episode report visiting any service provider for mental health reasons in the past year, and just 25.2% in low- and middle-income countries (LMIC) [2]. Fewer than half of those who sought help received minimally adequate treatment according to evidence-based guidelines [3].

The reasons for low demand for services in LMIC are poorly understood. The World Health Organization (WHO) advocates integrating evidence-based interventions into primary care to increase the availability and accessibility of services [4] as a strategy to reduce the gap. Yet in the World Mental Health Surveys, only 34.6% of people with depression in LMIC regarded themselves as needing treatment [3], suggesting that the treatment gap cannot be explained solely in terms of limited availability of mental health services.

Few data are available to inform strategies to promote treatment-seeking in LMIC, such as India, where the treatment gap for depression is over 85% [5]. Two recent systematic reviews on treatment-seeking for common mental disorders showed that “need factors”, such as greater symptom severity, chronicity, and disability, are positively associated with the likelihood of seeking treatment, and that women, the middle-aged, those with higher levels of education, and people of Caucasian ethnicity are more likely to seek treatment in high-income countries [6, 7]. They also showed that factors such as income, employment, and place of residence were generally not associated with treatment-seeking. However, there was a relative lack of evidence from LMIC, and few data were available with which to evaluate factors such as beliefs, attitudes, social support or health systems characteristics, which are hypothesised to be important to treatment decisions [7].

In India, data on treatment-seeking for depression are scarce. In 2016, a systematic review of “contact coverage” [8] (i.e. the proportion of adults with depression who sought treatment for depression [9]) found only one population-based study of treatment-seeking from India. This study reported that rural residents were less likely to seek treatment than urban residents, with no clear association with wealth [10]. However, the researchers used receipt of a depression diagnosis as its outcome measure, which conflates treatment-seeking with health care providers’ ability to detect and diagnose depression. Other Indian studies have reported on the use of general health services by people with depression, but without distinguishing between treatment sought for depression and for other health problems [11–13]. As such, very little evidence is available from India to inform efforts to reduce the treatment gap for depression.

Evidence-based strategies for reducing the treatment gap can only be devised if service planners have access to information on who seeks treatment, under what circumstances, and from where, some of which may differ between settings. This study is a descriptive analysis of treatment-seeking for depression by adults in Sehore sub-district, Madhya Pradesh, with the following specific objectives:

1. To estimate the proportion of adults who screen positive for depression who consult different types of treatment providers, (a) for depression symptoms and (b) for any reason, and to compare the latter with general health care use by people who screen negative for depression;
2. To measure the prevalence of self-reported barriers to using health services among adults who screen positive for depression;
3. To estimate the change in probability of treatment-seeking for symptoms of depression associated with need, predisposing and enabling factors, among screen-positive individuals.

## Methods

### Sample

This report is a secondary analysis of data from a population-based, cross-sectional community survey carried out with the primary aim of estimating the change in treatment-seeking among adults who screen positive for depression, before and after implementation of the MHCP. This secondary analysis focuses on characterising treatment-seeking patterns for screen-positive adults in both rounds.

The study design, sampling plan, and data collection for the original survey have been described in detail elsewhere [14, 15]. Briefly, data collection for the first round took place prior to Mental Health Care Plan implementation, in two waves (May–June 2013 and January–March 2014), and the second round after implementation of the plan (October–December 2016). The target population was adults (aged 18 and above) residing within the implementation area, with participants selected from voter lists through systematic random sampling. Inclusion criteria were fluency in spoken Hindi, residency in the selected household, willingness to provide informed consent, and absence of cognitive impairments that would preclude informed consent or ability to participate.

Across both rounds, 6203 adults were recruited, 6134 (98.9%) consented to participate, and 4297 resided within catchment areas of the de facto implementation area, where treatment was made available in the Community Health Centres. Of the 4297, 568 adults (289 in round 1, 279 in round 2) screened positive for depression and comprise the primary sub-sample for this secondary analysis. No difference in the probability of treatment-seeking for depression was observed between rounds, so for the purposes of the current analyses, data from both rounds were pooled to increase statistical power [16]. In order to compare use of health services by adults with and without depression, for this analysis we also included the 3531 community survey participants who resided within the implementation, did not screen positive for depression, and who did not report equivalent symptoms within the past 12 months. The sample size was calculated for the parent study based on the numbers required to detect a difference in contact coverage between rounds (the proportion of people with depression and alcohol use disorders who sought treatment for their condition), as described elsewhere [14].

### Setting

Sehore sub-district is a predominantly rural area in Madhya Pradesh, with a population of 427,432 [17]. 31.7% live below the poverty line and agriculture is the mainstay of the local economy [18]. General health indicators are below the national average [19], literacy rates are 81% for males and 58% for females [17], and 88% of residents have completed only primary education or less [20].

The PRIME programme (Programme for Improving Mental Health Care) aimed to implement and evaluate district-level Mental Health Care Plans (MHCP) [21]. The MHCP for Sehore focussed on depression, psychosis and alcohol use disorders and was implemented through community health centres between August 2014 and October 2016 [22].

Prior to implementation of the MHCP, public mental health services were mapped in detail, and major gaps were identified [23]. Outpatient and inpatient services were provided through Sehore District Hospital, by one psychiatrist and one clinical psychologist who are employed under the District Mental Health Programme and provide their services on alternate days, with periodic “outreach camps” [23]. No psychotropic medication or psychosocial interventions were available in primary care facilities, there were no psychiatric social workers or psychiatric nurses, and primary care workers were largely untrained in identifying and treating mental disorders.

After the plan was implemented, depression treatment was available at three Community Health Centres with psychological interventions delivered by case managers and pharmacological treatments prescribed for severe cases by medical officers. Community awareness activities were conducted to encourage service uptake, such as community meetings and proactive case finding in the community by the case managers. They also screened patients in Community Health Centres. The study area [23], Mental Health Care Plan [14], and PRIME evaluation plan [15] have been described in more detail elsewhere. The term “implementation area” will be used to refer to those villages where MHCP activities were fully implemented.

Although the extent to which private providers deliver mental health care was not assessed in the same way, other assessments of the wider health system in Madhya Pradesh show that private healthcare providers outnumber public providers [24, 25]. 67% of private health care providers in rural Madhya Pradesh have no formal medical qualifications [24]. Nonetheless, widespread dispensing of psychotropic medication has been documented by unqualified private practitioners [26, 27]. Quality of care in the private sector is low, as judged by correct diagnoses, following clinical checklists, and prescription of effective treatment, but has been found to be equally low among public providers [28]. There were no mental health specialists working in the private sector within the catchment area, but several were available in the nearest city, Bhopal.

Besides allopathic (biomedical) healthcare, a variety of traditional and religious healing systems are also available, including indigenous approaches such Ayurveda, yoga, naturopathy, Unani, Siddha, homoeopathy, and local systems of medicine, as well as spiritual remedies [29]. The parallel use of multiple systems has been frequently documented [30, 31].

### Data collection

Interviews were administered orally, in Hindi, by trained local fieldworkers who recorded participant responses using a questionnaire application programmed on Android tablets. Bias and data errors were minimised through extensive training, data validation rules in data entry, close supervision of data collectors, ongoing data checks in real time and corresponding feedback. The structured questionnaire included sections on socio-demographic details, health care use, barriers to using health services, depression symptoms, treatment-seeking for depression, alcohol use and related treatment, disability, internalised stigma related to depression and alcohol use, suicidal ideation and behaviours, and mental health knowledge and attitudes.

### Study measures

The Patient Health Questionnaire (PHQ-9) consists of 9 items on depression symptoms which are summed to generate a symptom score [32]. We used a cut-off point of ≥10 to indicate probable depression [33, 34] which has previously been validated in India [35, 36]. Participants were also asked if they had experienced equivalent symptoms for any 2 week period in the past 12 months.

Barriers to the use of health services were based on the Study on Global Ageing and Adult Health (SAGE) [37]. We added one question in round 2 on distance to health services. These barriers were not specific to depression.

We chose factors to investigate based the Andersen socio-behavioural model [38, 39], which groups factors associated with health service utilisation into; (a) need factors, which include both objective and subjective assessments of health status, (b) predisposing factors, covering both demographic characteristics and attitudinal factors such as health beliefs, and (c) enabling factors, which refers to structural determinants such as financial situation, transport and social support.

Predisposing factors included gender, religion, education, age, caste, marital status and internalised stigma (measured using questions from the Internalized Stigma of Mental Illness (ISMI) scale [40]). Enabling factors included land ownership, housing type, employment status, discussing depression symptoms with someone, and reporting cost and travel barriers to health care. Need factors included symptom severity, disability (measured using the 12-item World Health Organization Disability Assessment Schedule (WHODAS 2.0) [41]), perceived need for health care, probable alcohol use disorder (measured using the Alcohol Use Disorders Identification Test (AUDIT) with a cut-off of ≥8 [42–45]), suicidal thoughts (measured using the Composite International Diagnostic Interview (CIDI) suicidality module [46]), and PHQ-9 item-specific symptoms of depression.

Treatment-seeking was measured after completing the PHQ-9 questionnaire by asking “Did you seek any treatment for these problems at any time in the past 12 months?”. Thus, in this report, “treatment-seeking for depression” refers to seeking treatment for the symptoms listed in the PHQ-9. Participants who answered affirmatively were asked to specify the type of provider consulted (generalist, specialist, or traditional).

In the section on health care utilisation, participants were asked “In the last three months, have you visited any health facility or provider for any health problem?”, and if so, how many times. For each visit, participants were asked in which sector the consultation took place, and for what health condition (from a list of pre-defined options; see supplementary material). In this question, healthcare providers were divided into public, private, traditional, and mental health specialists. The outcome variable used for health care utilisation was whether a recent consultation had taken place (binary) and, if so, in which sector(s). Details of all measures used, and how these were treated in the analysis, are presented in the supplementary material.

### Analysis

First, we describe the sociodemographic and clinical characteristics of the sub-sample of adults with probable depression, using unweighted counts and weighted percentages to account for the sampling design.

To estimate the proportion of adults with probable depression who consult different types of treatment providers for depression symptoms and for general healthcare, we present the frequency of self-reported treatment-seeking for depression symptoms and general health care use, using weighted percentages and unweighted counts. We also present the frequency of general health care use by adults without depression (excluding those who reported depression symptoms over the past 12 months) and compare these proportions using Chi squared tests.

We next measure the prevalence of self-reported barriers to health service use by adults with probable depression, by presenting percentages on the frequency with which each barrier was reported, again using weighted percentages and unweighted counts.

To assess the association between perceived need, predisposing and enabling factors and treatment-seeking for depression, we present the proportion of adults with probable depression who sought treatment for depression by each characteristic, along with prevalence ratios and 95% confidence intervals, and tested the association between each variable with the outcome of treatment-seeking for depression using univariable log-linear regression analyses. For brevity, we present only the results for factors where this association reached a significance level of *p* < 0.05, but a full table is included in the supplementary material. Since these analyses were intended to be exploratory and hypothesis-generating, rather than causal and hypothesis-testing, we did not conduct multivariable analyses to control for potential confounders. In order to interpret the findings on the effect of discussing depression symptoms (presumed to be a proxy measure for social support), we also examined participants’ self-reports on who they discussed symptoms with, but the numbers in each group were too small to treat as separate variables.

All analyses were conducted using Stata/IC 15.1 [47]. Frequencies are reported as observed, while percentages, regression coefficients, 95% confidence intervals, and *P*-values are design adjusted. There were no missing data in the dataset.

### Ethics

Researchers explained the purpose of the survey to potential participants, read out the contents of study information sheets, and answered potential participants’ questions. Informed consent was indicated with either a signature or a thumbprint. All screen-positive participants who were not receiving treatment were referred to the nearest public health facility where depression treatment was available.

Ethical approval was provided by the World Health Organization Research Ethics Review Committee (Geneva, Switzerland), the Sangath Institutional Review Board (Goa, India), and the London School of Hygiene & Tropical Medicine Observational Ethics Committee (London, United Kingdom) (ref: 10439).

### Research paradigm

This study forms part of a larger mixed methods research project that uses a pragmatic approach [48]. However, the current study assumes a predominantly realist ontology, and applies an empiricist epistemology to address research questions posed within this paradigm. In terms of axiology, we start from the value judgement that human flourishing is inhibited by experiences of depression symptom, and that it is intrinsically worthwhile to alleviate the suffering that arises from these experiences where possible.

## Results

### Sample characteristics

The socio-demographic and mental health characteristics of participants with and without probable depression are described in Table 1. Among those with probable depression, the mean age was 45.4 years, there were approximately equal proportions of men and women (53.8% female), and most participants were Hindu (92.1%), married (81.7%), and had not completed primary education (74.1%). The majority of participants with probable depression had moderate symptoms (77.9%). Tiredness or lack of energy was the most frequently reported symptom (reported by 79.2% on more than 7 days in the past 2 weeks), followed by feeling depressed or hopeless (63.3%).

**Table 1:** Socio demographic and mental health characteristics of adults with and without probable depression in Sehore sub-district, India, 2013–2016

Characteristic	Adults with probable depression, N (%)	Adults without probable depression, N (%)
Gender		
*Female*	321 (53.8)	1589 (43.9)
*Male*	247 (46.2)	1942 (56.1)
Age group, years		
*18–29*	98 (17.5)	1138 (32.8)
*30–49*	248 (44.1)	1514 (43.0)
*50–90*	222 (38.4)	879 (24.2)
Education level completed		
*Less than primary*	419 (74.1)	2017 (56.8)
*Primary*	129 (22.4)	1124 (32.8)
*Secondary or more*	20 (3.5)	390 (10.5)
Employment status		
*Unemployed*	20 (4.2)	53 (1.7)
*Productive non-income*	241 (38.5)	1401 (34.0)
*Low income*	277 (51.9)	1785 (52.4)
*High income*	30 (5.4)	289 (7.9)
Religion		
*Hindu*	525 (92.1)	3180 (89.8)
*Muslim*	43 (7.9)	350 (10.2)
*Christian*	0 (0)	1 (0.0)
Caste		
*Scheduled Caste*	101 (15.8)	516 (14.2)
*Scheduled Tribe*	25 (4.2)	140 (4.0)
*Other Backwards Caste*	393 (71.0)	2503 (71.1)
*General*	49 (9.1)	372 (10.7)
Marital status		
*Single*	32 (6.4)	375 (10.9)
*Married*	461 (81.7)	2953 (83.9)
*Widowed/Separated/Divorced*	75 (11.9)	213 (5.3)
Current depression severity (PHQ-9 score)		
*Moderate* (10–14)	450 (77.9)	0 (0.0)
*Moderately severe* (15–19)	107 (20.1)	0 (0.0)
*Severe (≥20)*	11 (2.0)	0 (0.0)
Depression-related symptoms reported on more than 7 days in past 2 weeks		
*Tiredness/lack of energy*	450 (79.2)	842 (23.3)
*Feeling depressed or hopeless*	371 (63.3)	255 (7.5)
*Sleep problems*	333 (58.1)	347 (9.8)
*Lack of interest or pleasure*	289 (53.3)	175 (5.5)
*Appetite problems*	293 (49.9)	282 (8.2)
*Lack of concentration*	229 (40.3)	155 (4.5)
*Low self-esteem/feeling like a failure*	123 (22.5)	38 (1.2)
*Slow movements/restlessness*	119 (22.2)	47 (1.3)
*Thoughts of death/self-harm*	37 (7.4)	3 (0.0)

Counts reported as observed, percentages are design adjusted

The non-depressed group included more males, more people with secondary education, fewer unemployed people, and the mean age was lower (39.6 years).

### Objective 1: use of health services and treatment-seeking for depression

Table 2 shows the health care used for any reason in the past 3 months by adults with and without probable depression.

**Table 2:** Health care used in the past 3 months for any reason by adults with and without probable depression in Sehore sub-district, 2013–2016

	By those with probable depression, N (%) (***n*** = 568)	By those without probable depression, N (%) (***n*** = 3531)	***P***-value
Private health care provider	165 (32.0)	638 (19.0)	< 0.0001
Public health care provider	108 (19.6)	408 (11.5)	< 0.0001
Traditional service provider	119 (19.2)	675 (18.2)	0.60
Mental health specialist	1 (0.3)	0 (0)	0.02
Other	3 (0.6)	5 (0.2)	0.06
None	205 (34.4)	1909 (54.3)	< 0.0001

Counts reported as observed, percentages are design adjusted

65.6% of adults with probable depression had used health services for some reason in the past three months. Of these, 48.4% consulted the private sector while 29.8% consulted public providers and 29.3% consulted traditional providers. Those with probable depression were more likely to have used health services in the past 3 months than those without depression (65.6% vs. 45.7%, *p* < 0.0001).

Table 3 shows treatment sought specifically for depression symptoms in the past 12 months by adults with probable depression.

**Table 3:** Health care used in the past 12 months for depression symptoms by adults with probable depression in Sehore sub-district, 2013–2016

	By those with probable depression, N (%) (***n*** = 568)
Generalist health worker (including case managers employed under the mental health care plan)	48 (8.5)
Specialist mental health worker	13 (3.1)
Traditional service provider	14 (2.3)
None	493 (86.1)

Counts reported as observed, percentages are design adjusted

Total exceeds 100% because some participants visited more than one sector

13.9% of adults with probable depression sought treatment for depression symptoms, and of these, 61.3% did so from generalist providers, compared to 22.1% who consulted specialists and 16.7% who consulted traditional service providers.

1.9% of adults with probable depression who sought health care reported that any of their consultations were for depression or anxiety-related problems, and 1.4% reported that any consultation was for other mental health-related problems.

### Objective 2: barriers to health care use

Table 4 presents self-reported barriers to health care use by adults with probable depression. Cost and distance barriers were the most commonly reported barriers, with each reported by more than half of the sample who were asked about these (54.3 and 52.3%, respectively). The third most commonly reported barrier was the belief that health services were not needed (31.3%).

**Table 4:** Self-reported barriers to health care use among adults with probable depression in Sehore sub-district, 2013–2016

Barrier	Number of adults with probable depression who reported barrier (%)
Fees are not affordable	302 (54.3)
Services are too far away	145^a^(52.3)
Services not currently needed	172 (31.3)
Dislike taking medications	178 (30.9)
Care received is not good enough	148 (26.2)
Care providers do not understand my health problems	135 (23.4)
They don’t have medicines I need	95 (17.3)
They frequently run out of medicines	81 (15.9)
Other reason	56 (11.2)
Seeking some kinds of treatment can make me or my family feel embarrassed	38 (8.1)

All percentages are adjusted for the complex sampling strategy

^a^only measured in follow up round, so denominator was 279

### Objective 3: factors associated with treatment-seeking for depression symptoms

Table 5 shows those associations between need, predisposing and enabling factors and treatment-seeking among all adults with probable depression that evidence suggestive of an association (*P* < 0.05). See the supplementary material for the full set of results.

**Table 5:** Association between need, predisposing and enabling factors and treatment-seeking for depression among adults with probable depression in Sehore sub-district, 2013–2016

	Total seeking treatment (n)	Prevalence of treatment-seeking, % (95% CI)	Prevalence ratio (95% CI)	***p***-value
**Need factors**				
Symptom severity (total current PHQ score)				
*10–14*	50/450	11.5 (8.5–15.5)	1	< 0.01
*15–19*	20/107	20.7 (13.2–30.8)	1.79 (1.11–2.88)	
*≥ 20*	5/11	39.5 (12.8–74.5)	3.42 (1.33–8.81)	
Tiredness/lack of energy				
*< 7 days in past 2 weeks*	10/118	7.3 (3.8–13.5)	1	0.03
*≥ 7 days in past 2 weeks*	65/450	15.7 (11.7–20.6)	2.14 (1.08–4.24)	
Lack of interest or pleasure				
*< 7 days in past 2 weeks*	26/279	9.7 (6.3–14.7)	1	0.01
*≥ 7 days in past 2 weeks*	49/289	17.6 (13.2–23.2)	1.82 (1.16–2.85)	
Low self-esteem / feeling like a failure				
*< 7 days in past 2 weeks*	51/445	11.5 (8.5–15.3)	1	< 0.01
*≥ 7 days in past 2 weeks*	24/123	22.4 (15.2–31.9)	1.96 (1.28–3.00)	
Slow movements / restlessness				
*< 7 days in past 2 weeks*	51/449	12.2 (8.9–16.5)	1	0.01
*≥ 7 days in past 2 weeks*	24/119	20.1 (14.4–29.3)	1.65 (1.13–2.39)	
**Predisposing factors**				
Marital status				
*Single / separated / widowed*	7/107	5.9 (2.7–12.2)	1	0.02
*Married*	68/461	15.7 (11.9–20.6)	2.67 (1.19–5.99)	
**Enabling factors**				
Spoken to someone about these problems				
*No*	13/352	3.9 (2.2–7.0)	1	< 0.001
*Yes*	62/216	29.4 (23.1–36.5)	7.50 (4.11–13.68)	
Services don’t have medications I need				
*No*	55/473	11.9 (8.7–16.2)	1	0.01
*Yes*	20/95	24.4 (15.9–35.6)	1.99 (1.19–3.32)	
Services frequently run out of medications				
*No*	56/487	11.9 (8.4–16.7)	1	0.01
*Yes*	19/81	23.6 (16.3–33.0)	2.05 (1.23–3.39)	

Counts reported as observed. Prevalence ratios, percentages and *P*-values are design adjusted

This table presents data for only those factors for which there was evidence suggestive of an association with treatment-seeking for depression (P < 0.05). See the supplementary material for full set of findings

Among the “need factors”, the following were positively associated with treatment-seeking: symptom severity (39.5% of those with severe symptoms sought treatment compared to 11.5% of those with moderate symptoms), and reporting four specific symptoms on the PHQ-9 on 7 or more days in the past 2 weeks; tiredness or lack of energy, lack of interest or pleasure, low self-esteem or feeling like a failure, and slow movements or restlessness.

Under “predisposing factors”, 5.9% of unmarried people (single, separated or widowed) sought help for depression compared to 15.7% of those who were married.

Among “enabling factors”, 29.4% of those who discussed symptoms sought help compared to 3.9% of those who did not. Spouses were the most common person who symptoms were discussed with (67.5%; data not presented). There was a positive association between treatment-seeking for depression and reporting that “services frequently run out of medications” and “services don’t have the medications I need” as barriers.

## Discussion

### Principal findings

Although few people sought treatment specifically for depression symptoms, almost two thirds of adults with probable depression had recent contact with health services, which was significantly higher than by adults without probable depression. The private sector was most frequently consulted, while traditional services were used least, indicating that private health services are an important platform through which individuals with depression could theoretically be identified and treated. Structural barriers to using health services such as cost and distance are felt to be major barriers to the use of health care, but the current evidence suggests that reporting these barriers is unrelated to treatment-seeking for depression. These findings suggest the potential importance of social support and marriage in seeking treatment for depression in this context.

### Strengths and limitations

To our knowledge, this study is the most comprehensive population-based study to explore patterns of treatment-seeking for depression in India. The current study used a large, representative community-based sample, to show which groups should be targeted in order to reduce the treatment gap for depression. These results are likely to have high external validity in other rural Indian settings, given the minimal exclusion criteria applied. Given the dearth of research on this topic from LMIC, the current findings may provide useful insights for service planning and policy, and generate hypotheses about barriers to treatment-seeking for further testing.

Since this was a secondary analysis of data collected for another primary purpose, however, we were limited by the measures used. More detailed, mental health-specific measures of barriers to care exist that were not employed due to interview length considerations, such as the Barriers to Access to Care Evaluation scale (BACE) [49]. This limits the extent to which our results can be compared to recent studies from other settings (e.g. [50]), which could help to distinguish context-specific from more universal barriers. The measures used to indicate economic status are also imperfect proxies, meaning that we cannot be sure from our findings that poverty does not inhibit treatment-seeking for depression, despite the lack of association found here. Equally, the sample size was determined with reference to the primary aim of the parent study, and as such some of the current analyses may have been under-powered to detect an association, particularly for rare characteristics such as unemployment and suicidal thoughts.

Participants’ mental health status was determined using a screening tool, not full diagnostic interviews, so the sample is likely to include some false positives, especially given the low positive predictive value of the PHQ-9 reported in Goa [35]. Furthermore, since these data were generated through a cross-sectional survey, symptom severity, level of disability and attitudes towards health services were measured only at the time of the interview despite being subject to change over time, whereas treatment-seeking was measured retrospectively over the past 12 months.

There is also the potential for non-response bias, since only 62.5% of selected adults were located at baseline, and 76.2% at endline, due to death or migration. If those who were not located differ systematically from those who were, this would result in biased estimates. We have no data on those who were not located, although our sample characteristics are generally comparable to the most recent census data [17].

Finally, self-reported data are always potentially open to social desirability bias, especially when using face-to-face interviews, and it is possible that this led to under-reporting of traditional service use. However, our estimates are in line with a recent national survey showing that the use of traditional healers is low relative to the use of allopathic care for both minor and major morbidity, even in rural areas [51].

### Implications for service planning and future research

#### Use of health services for non-depression reasons

Adults with high levels of depression symptoms are likely to be in contact with health services, but their primary complaints are rarely the depression symptoms listed in the PHQ-9. This echoes previous findings from India that depressed individuals frequently present to health services with somatic symptoms [11, 12, 52–54]. Therefore, the most important challenge from a public health perspective appears not to be to persuade depressed individuals to visit services, but rather to enable health workers to recognise their mental health needs during consultations about other complaints. In other words, the relevant “treatment gap” is not between those who do and do not consult health services, but between those who receive effective treatment and those who do not. Health workers should be trained and supervised to distinguish psychosomatic symptoms from other health problems that are comorbid with depression, and provide appropriate care.

#### Use of the private sector

Importantly, however, we found that adults with depression are more likely to consult private than public health care providers, highlighting the importance of engaging private providers in initiatives to improve depression care. In the state of Madhya Pradesh, 76% of qualified medics and 72% of qualified paramedical staff are employed in the private sector [24]. India has one of the most privatised health systems in the world [25], with around 80% of outpatient care provided in the private sector [55, 56]. High rates of private health care use have been linked to the underfunding and poor performance of the public health sector [25], and public perceptions that public services are of poor quality [57].

Interventions delivered through the public health system have little chance of reducing the treatment gap in a context where the majority of health care consultations take place elsewhere. The current landscape of the Indian health system is not reflected in the Global Mental Health literature, where traditional services are often discussed [58–64], but private providers are rarely mentioned, despite evidence that they frequently dispense psychotropic medications in India [65]. The MANAS trial in Goa demonstrated the feasibility of training and supervising private providers to strengthen their ability to detect and treat depressive disorders [66]; this strategy should be evaluated in other regions of India.

#### Use of traditional services

We also found that only a small proportion of treatment sought by people with probable depression was in the traditional sector. The report of the 2015–16 National Mental Health Survey of India posits preference for traditional services as a major barrier to the use of formal treatment [5], based on qualitative interviews with health professionals and community leaders, but presents no quantitative data on service use. Common mental disorders were not distinguished from severe mental illness in these interviews, so it is possible that the difference between our results and the perceptions of these stakeholders arose because traditional providers play an important role in treating people with psychotic disorders but not depression. Our estimates are backed up by a recent national survey showing that the use of traditional healers is low relative to the use of allopathic care for all health conditions, even in rural areas [51]. This suggests that engaging with or influencing the use of traditional services should not be a major policy focus in improving care for depression in this context.

#### Detecting depression: symptomatology and help-seeking

In terms of improving detection of depression in health services, health workers should be aware that tiredness or lack of energy is the most common symptom reported by depressed people in this population, followed by depressed mood or hopelessness. Those experiencing lack of energy are more likely to present to health services with depression symptoms than those with depressed mood, potentially because the former symptom is seen as a more legitimate medical complaint than emotional symptoms [67]. Future research should test the predictive value of brief questions using local idioms of distress, as in recent research in Nepal [68], to find the most efficient way of detecting depression among primary care attendees who present with somatic symptoms, during short consultations [69].

#### Factors associated with treatment-seeking for depression

It is important to understand why people with probable depression in rural India do not generally seek health care for depression symptoms per se (when “depression symptoms” are conceptualised as those listed in the PHQ-9 questionnaire) in order to appropriately interpret and respond to the treatment gap for depression. As discussed above, one possibility is that individuals with depression frequently express their distress through somatic rather than psychological symptoms [11, 12, 52–54], which are not captured by the PHQ-9. This has important implications for how the treatment gap is measured and conceptualised in future studies in comparable settings. Our recent qualitative study found that psychological symptoms of depression are rarely considered to be medical conditions in this context [70].

The current findings on factors associated with treatment-seeking for depression symptoms provide tentative evidence on the characteristics that distinguish those who do seek depression care from those who do not. These should be interpreted with caution, since these analyses were exploratory and hypothesis-generating rather than hypothesis-testing: The results show which groups seek treatment rather than establishing causal relationships between these factors. However, some intriguing hypotheses were generated that deserve further investigation about why some groups may be more likely to seek help for their psychological symptoms than others.

Firstly, we found that people with more severe depression symptoms were more likely to report having sought treatment for these symptoms, which is consistent with previous evidence from other settings [7, 71]. It is possible that people with milder symptoms do not consider their problem to be severe enough to warrant formal care. In support of this hypothesis, evidence from the World Mental Health Surveys [72] and from a large dataset from the USA [73] both showed that lack of perceived need for treatment and preferring to handle the problem themselves are the most common barriers to treatment-seeking, indicating that demand for mental health treatment is low among mild- to moderate cases. In both cases those with more severe symptoms were less likely to say that they had no need for services.

The current evidence did not support an association between treatment-seeking and disability or perceived need for health care, however, which is at odds with international evidence [7]. In light of the high rates of general health service use, this may be because people with depression consider themselves to have other health problems, and attribute their disability and associated need for health care to these non-depression symptoms. Future research should assess the overall health needs of adults with depression and investigate the effect of comorbid conditions on help-seeking behaviour.

Secondly, we found no evidence that those with lower levels of self-stigma, exposure to mental health communications, or indicators of higher mental health literacy were more likely to seek treatment for depression. This contrasts with the conclusions of previous Indian studies [13, 74]. This may be because in the current study research workers referred to specific symptoms of depression, rather than to mental illness or psychiatric treatment, and some evidence suggests that symptoms of common mental disorders are not considered to represent mental illness in India [53]. Further research is needed to test this interpretation, especially in light of the current absence of evidence that anti-stigma or awareness campaigns effectively alter treatment-seeking behaviour for common mental disorders [75].

Thirdly, while a majority of participants felt that cost and distance barriers are important, in line with previous research [13, 74, 76], those who reported these barriers were no less likely to seek treatment for depression symptoms. We also found little evidence to support differences in treatment-seeking by socio-economic status, which echoes findings from the World Mental Health Surveys [77]. This requires further investigation, both to explicitly test the hypothesis that treatment-seeking for depression symptoms (at least when measured as a binary variable, without differentiating between sectors or provider types) is not associated with key enabling factors such as economic and geographical constraints, and to qualitatively explore why this is so, should this finding be replicated.

Intriguingly, the gender differences often reported in studies from high-income countries were not replicated in this setting, and we found the opposite association between marital status and treatment-seeking from that which is typically reported elsewhere [6, 7, 78]. This suggests the importance of local data in identifying vulnerable groups for service planning, and provides tentative evidence that processes believed to inhibit treatment-seeking in other cultural contexts – such as masculine ideals of self-sufficiency [79, 80] – may not apply in the same way to Indian populations. Again, of course, this finding should be replicated before it used to inform service planning.

Finally, participants who reported limited availability or irregular supply of medications were counter-intuitively more likely to seek treatment for depression than those who did not report these barriers. This may result from retrospective measurement of these factors, since negative experiences of health care affect attitudes towards services [81]. Longitudinal studies are needed to establish causal relationships between attitudes to services and help-seeking behaviour, and test the impact of negative experiences of health care on subsequent attitudes and treatment-seeking behaviour.

Future research should test the hypotheses generated here while controlling for confounding factors, and investigate factors for which data were not available including contextual influences such as social norms. Qualitative research is important to identify factors that the community perceives to be important, and to better understand why so few adults with probable depression consider treatment to be necessary for these symptoms specifically.

## Conclusion

Although most participants had not sought help specifically for depression symptoms, almost two thirds reported some recent contact with health services, most frequently in the private sector. Private health care providers are likely to be an important group to engage in efforts to improve detection and treatment of depression in this area, and their inclusion should be considered in programmes of training and supervision to reduce the treatment gap for depression. Future research should investigate why adults with probable depression seek help primarily for non-depression symptoms, and seek to replicate the current findings on factors associated with treatment-seeking for depression symptoms.

## Supplementary information

**Additional file 1: Table 6.** Instruments used to measure barriers to health service use and factors associated with treatment-seeking for depression in PRIME community survey, Sehore sub-district, India, 2013–2016. **Table 7.** Association between need, predisposing and enabling factors and treatment-seeking for depression among adults with probable depression in Sehore sub-district, 2013–2016 – all factors.

## Data Availability

The datasets analysed during the current study are available from the PRIME consortium on reasonable request. See PRIME website for details and to request access: http://www.prime.uct.ac.za/. Queries regarding access to the data can be directed to the PRIME project manager, Erica Breuer: erica.breuer@uct.ac.za. The PRIME community survey questionnaire that was used here has been previously published elsewhere (see e.g. Rathod et al., 2016, [21]).

## Abbreviations

AUDIT: Alcohol Use Disorders Identification Test
BACE: Barriers to Access to Care Evaluation scale
CIDI: Composite International Diagnostic Interview
ISMI: Internalized Stigma of Mental Illness scale
LMIC: Low- and middle-income countries
MHCP: Mental Health Care Plan
PHQ-9: Patient Health Questionnaire – 9 item version
PRIME: Programme for Improving Mental Health Care
SAGE: Study on Global Ageing and Adult Health
WHO: World Health Organization
WHODAS: World Health Organization Disability Assessment Schedule
