# Supplementary material for: Health care use and treatment-seeking for depression symptoms in rural India: an exploratory cross-sectional analysis
Source: BMC Health Serv Res. 2020 Apr 6;20:287. doi: 10.1186/s12913-020-05162-0 (PMC7137455; doi:10.1186/s12913-020-05162-0)
Supplement: Supplementary file 1 — Additional file 1: Table 6. Instruments used to measure barriers to health service use and factors associated with treatment-seeking for depression in PRIME community survey, Sehore sub-district, India, 2013–2016. Table 7. Association between need, predisposing and enabling factors and treatment-seeking for depression among adults with probable depression in Sehore sub-district, 2013–2016 – all factors. [file 12913_2020_5162_MOESM1_ESM.docx]

## Supplementary material

**Table 6.** Instruments used to measure barriers to health service use and factors associated with treatment-seeking for depression in PRIME community survey, Sehore sub-district, India, 2013-2016.

| **Factor** | **Instrument** | **Categories used for analysis** |
| --- | --- | --- |
| Symptom severity and specific symptoms | Patient Health Questionnaire (PHQ-9) (Kroenke et al. 2001) | Total score (sum of items): None (0-4)/ Mild (5-9)/ Moderate (10-14)/ Moderately severe (15-19) /Severe (≥20)  Individual symptoms: ≥7 days in past 2 weeks/<7 days in past 2 weeks |
| Disability | 12-item World Health Organization Disability Assessment Schedule (WHODAS 2.0) (Üstün et al. 2010) | Total score (complex scoring, divided into terciles) |
| Probable alcohol use disorder | Alcohol Use Disorders Identification Test (AUDIT) (Babor et al. 2001; Carey et al. 2003; Pal et al. 2004; de Meneses-Gaya et al. 2009) | Screen-positive (AUD≥8, AUD<8) |
| Suicidal thoughts | Composite International Diagnostic Interview (CIDI) suicidality module (Kessler and Üstün, 2004) | Yes/No |
| Barriers to healthcare use (including lack of perceived need) | Questions from the Study on global AGEing and adult health (SAGE) (Kowal et al. 2012), with one question added in round 2 on distance to services | Agree or strongly agree / disagree or strongly disagree |
| Self-stigma | Questions from the Internalized Stigma of Mental Illness (ISMI) scale (Boyd et al. 2014) | Total score: Sum of items (divided into terciles) |
| Land ownership | No structured instrument used | Yes/ No |
| Housing type | No structured instrument used | Kuccha (lowest level)/ Pucca (highest level)/ Semi-Pucca (mid-level) |
| Employment status | No structured instrument used | Unemployed/ Productive non-income (students and housewives)/ Low income/ High income |
| Discussing depression symptoms | No structured instrument used | Yes/ No  If yes: Friend / Neighbour / Spouse / Parents / Siblings / Relatives / Employer or coworker / Other |
| Gender | No structured instrument used | Male/ Female |
| Religion | No structured instrument used | Hindu / Muslim / Christian / Sikh / Buddhist / Jain / Other |
| Education level | No structured instrument used | Less than primary school completed / Primary school or more completed |
| Age | No structured instrument used | 18-29/ 30-49/ 50-90 |
| Caste | No structured instrument used | General/ Scheduled caste/ Scheduled tribe/ “Other Backward Caste” |
| Marital status | No structured instrument used | Single/ Ever married |
| Health care utilization | Questions from the Client Socio-Demographic and Service Receipt Inventory (CSSRI) (Chisholm et al. 2000) | Any use of health care in the past 3 months (private/ public/ traditional/ other/ none) |
| Treatment-seeking for depression | No structured instrument used | Overall: Yes/ No  Provider type: Specialist mental health workers (psychiatrists, other specialist medics, psychologists, counsellors, psychiatric nurses, other mental health professionals) / Generalist health workers (other medical doctor, social worker, community health worker, nurse, ANM, ASHA, AWW, PRIME case managers based in primary care facilities*), Complementary service providers (ojha, guni, dev maharaj, traditional healers, herbalists, spiritualists).  *available in round 2 only |

**Table 7.** Association between need, predisposing and enabling factors and treatment-seeking for depression among adults with probable depression in Sehore sub-district, 2013-2016 – all factors

|  | **Total seeking treatment (n)** | **Prevalence of treatment-seeking,**  **% (95% CI)** | **Prevalence ratio (95% CI)** | **p-value** |
| --- | --- | --- | --- | --- |
| **Need factors** | | | | |
| Symptom severity (total current PHQ score) |  |  |  |  |
| *10-14* | 50/450 | 11.5 (8.5-15.5) | 1 | <0.01 |
| *15-19* | 20/107 | 20.7 (13.2-30.8) | 1.79 (1.11-2.88) |  |
| *≥20* | 5/11 | 39.5 (12.8-74.5) | 3.42 (1.33-8.81) |  |
| Disability (total WHO-DAS score) |  |  |  |  |
| *Low* | 2/36 | 8.3 (1.7-31.7) | 1 | 0.15 |
| *Medium* | 14/148 | 9.9 (4.5-20.3) | 1.19 (0.23-6.21) |  |
| *High* | 59/384 | 15.9 (12.4-20.2) | 1.92 (0.44-8.32) |  |
| Perceived need for health care |  |  |  |  |
| *No* | 15/172 | 9.2 (4.5-18.1) | 1 | 0.12 |
| *Yes* | 60/396 | 16.1 (12.4-20.5) | 1.74 (0.86-3.54) |  |
| Comorbid AUD |  |  |  |  |
| *No* | 69/531 | 13.7 (10.6-17.5) | 1 | 0.53 |
| *Yes* | 6/37 | 17.2 (7.5-34.7) | 1.25 (0.62-2.54) |  |
| Suicidal thoughts |  |  |  |  |
| *No* | 49/421 | 12.7 (9.4-16.9) | 1 | 0.21 |
| *Yes* | 26/147 | 17.2 (11.0-25.8) | 1.35 (0.84-2.16) |  |
| Sleep problems |  |  |  |  |
| *< 7 days in past 2 weeks* | 33/235 | 15.2 (10.5-21.4) | 1 | 0.60 |
| *≥ 7 days in past 2 weeks* | 42/333 | 13.0 (8.6-19.3) | 0.86 (0.49-1.51) |  |
| Tiredness/lack of energy |  |  |  |  |
| *< 7 days in past 2 weeks* | 10/118 | 7.3 (3.8-13.5) | 1 | 0.03 |
| *≥ 7 days in past 2 weeks* | 65/450 | 15.7 (11.7-20.6) | 2.14 (1.08-4.24) |  |
| Appetite problems |  |  |  |  |
| *< 7 days in past 2 weeks* | 37/275 | 14.8 (10.0-21.4) | 1 | 0.58 |
| *≥ 7 days in past 2 weeks* | 38/293 | 13.0 (9.6-17.5) | 0.88 (0.56-1.39) |  |
| Lack of concentration |  |  |  |  |
| *< 7 days in past 2 weeks* | 43/339 | 13.0 (9.7-17.2) | 1 | 0.50 |
| *≥ 7 days in past 2 weeks* | 32/229 | 15.3 (9.9-22.9) | 1.18 (0.73-1.89) |  |
| Lack of interest or pleasure |  |  |  |  |
| *< 7 days in past 2 weeks* | 26/279 | 9.7 (6.3-14.7) | 1 | 0.01 |
| *≥ 7 days in past 2 weeks* | 49/289 | 17.6 (13.2-23.2) | 1.82 (1.16-2.85) |  |
| Feeling depressed or hopeless |  |  |  |  |
| *< 7 days in past 2 weeks* | 32/197 | 16.8 (11.7-23.5) | 1 | 0.13 |
| *≥ 7 days in past 2 weeks* | 43/371 | 12.2 (8.9-16.7) | 0.73 (0.49-1.10) |  |
| Low self-esteem / feeling like a failure |  |  |  |  |
| *< 7 days in past 2 weeks* | 51/445 | 11.5 (8.5-15.3) | 1 | <0.01 |
| *≥ 7 days in past 2 weeks* | 24/123 | 22.4 (15.2-31.9) | 1.96 (1.28-3.00) |  |
| Slow movements / restlessness |  |  |  |  |
| *< 7 days in past 2 weeks* | 51/449 | 12.2 (8.9-16.5) | 1 | 0.01 |
| *≥ 7 days in past 2 weeks* | 24/119 | 20.1 (14.4-29.3) | 1.65 (1.13-2.39) |  |
| Thoughts of death / self-harm |  |  |  |  |
| *< 7 days in past 2 weeks* | 64/531 | 13.0 (9.5-17.6) | 1 | 0.11 |
| *≥ 7 days in past 2 weeks* | 11/37 | 25.3 (12.0-45.7) | 1.95 (0.87-4.37) |  |
| **Predisposing factors** | | | | |
| Sex |  |  |  |  |
| *Male* | 41/247 | 16.1 (11.3-22.3) | 1 | 0.22 |
| *Female* | 34/321 | 12.1 (8.4-17.1) | 0.75 (0.47-1.19) |  |
| Religion |  |  |  |  |
| *Hindu* | 68/525 | 14.1 (10.7-18.3) | 1 | 0.76 |
| *Muslim* | 7/43 | 12.2 (4.8-27.9) | 0.87 (0.34-2.19) |  |
| Education level completed |  |  |  |  |
| *Less than primary* | 51/419 | 12.9 (9.8-16.8) | 1 | 0.26 |
| *Primary or more* | 24/129 | 16.8 (10.5-25.9) | 1.30 (0.82-2.07) |  |
| Age |  |  |  |  |
| *18-29 years* | 9/98 | 9.2 (4.8-17.0) | 1 | 0.13 |
| *30-49 years* | 36/248 | 15.1 (10.7-20.8) | 1.64 (0.82-3.28) |  |
| *≥50 years* | 30/222 | 14.7 (10.2-20.8) | 1.60 (0.97-2.64) |  |
| Caste |  |  |  |  |
| *Scheduled caste (SC)* | 13/101 | 15.2 (8.1-26.7) | 1 | 0.62 |
| *Scheduled tribe (ST)* | 3/25 | 16.9 (6.0-39.1) | 1.11 (0.34-3.64) |  |
| *“Other backward caste” (OBC)* | 53/393 | 13.8 (9.7-19.1) | 0.91 (0.44-1.85) |  |
| *General* | 6/49 | 11.6 (5.4-23.3) | 0.77 (0.28-2.13) |  |
| Marital status |  |  |  |  |
| *Single / separated / widowed* | 7/107 | 5.9 (2.7-12.2) | 1 | 0.02 |
| *Married* | 68/461 | 15.7 (11.9-20.6) | 2.67 (1.19-5.99) |  |
| Heard about mental health in past 12 months |  |  |  |  |
| *No* | 22/191 | 12.4 (8.1-18.6) | 1 | 0.51 |
| *Yes* | 53/377 | 14.9 (10.5-20.8) | 1.20 (0.69-2.07) |  |
| Agree that medications can be effective for mental health problems |  |  |  |  |
| *Yes* | 59/464 | 13.9 (10.6-18.1) | 1 | 0.56 |
| *Don’t know* | 8/34 | 19.8 (9.4-37.1) | 1.43 (0.67-3.06) |  |
| *No* | 7/66 | 9.8 (4.0-21.8) | 0.70 (0.32-1.56) |  |
| Agree that people with mental health problems can recover |  |  |  |  |
| *Yes* | 53/368 | 15.9 (11.6-21.6) | 1 | 0.08 |
| *Don’t know* | 12/86 | 13.4 (7.4-23.0) | 0.84 (0.47-1.51) |  |
| *No* | 10/110 | 9.1 (5.1-15.8) | 0.57 (0.30-1.10) |  |
| Seeking services makes me feel ashamed or embarrassed |  |  |  |  |
| *No* | 69/530 | 13.5 (10.1-17.9) | 1 | 0.40 |
| *Yes* | 6/38 | 18.4 (9.3-33.3) | 1.36 (0.66-2.82) |  |
| Dislike taking medications |  |  |  |  |
| *No* | 60/390 | 16.1 (12.1-21.2) | 1 | 0.09 |
| *Yes* | 15/178 | 9.0 (4.7-16.6) | 0.56 (0.28-1.10) |  |
| Self-stigma |  |  |  |  |
| *Low* | 24/203 | 12.2 (7.4-19.3) | 1 | 0.41 |
| *Medium* | 23/191 | 14.0 (9.6-20.0) | 1.15 (0.65-2.04) |  |
| *High* | 28/174 | 15.6 (10.5-22.4) | 1.28 (0.71-2.30) |  |
| **Enabling factors** | | | | |
| Owning land |  |  |  |  |
| *No* | 46/402 | 12.3 (8.9-16.6) | 1 | 0.10 |
| *Yes* | 29/166 | 17.7 (12.0-25.3) | 1.44 (0.94-2.22) |  |
| House type |  |  |  |  |
| *Kuccha* | 35/309 | 12.8 (8.8-18.1) | 1 | 0.93 |
| *Semi-pucca* | 17/85 | 21.0 (12.6-33.0) | 1.65 (0.99-2.76) |  |
| *Pucca* | 23/174 | 12.5 (7.9-19.2) | 0.98 (0.55-1.75) |  |
| Employment |  |  |  |  |
| *Unemployed* | 1/20 | 5.4 (0.8-28.5) | 1 | 0.09 |
| *Productive non-income (student/housewife)* | 28/241 | 12.3 (8.2-18.0) | 2.28 (0.34-15.08) |  |
| *Low income* | 42/277 | 15.6 (11.2-21.4) | 2.89 (0.42-19.99) |  |
| *High income* | 4/30 | 15.9 (6.6-33.5) | 2.96 (0.58-14.92) |  |
| Fees not affordable |  |  |  |  |
| *No* | 32/266 | 12.7 (7.5-20.8) | 1 | 0.60 |
| *Yes* | 43/302 | 14.9 (11.2-19.8) | 1.18 (0.64-2.16) |  |
| Services too far away |  |  |  |  |
| *No* | 9/134 | 6.8 (3.8-12.0) | 1 | 0.24 |
| *Yes* | 16/145 | 10.5 (6.6-16.3) | 1.54 (0.74-3.19) |  |
| Spoken to someone about these problems |  |  |  |  |
| *No* | 13/352 | 3.9 (2.2-7.0) | 1 | <0.001 |
| *Yes* | 62/216 | 29.4 (23.1-36.5) | 7.50 (4.11-13.68) |  |
| Quality of services is not good enough |  |  |  |  |
| *No* | 56/420 | 14.2 (10.0-19.8) | 1 | 0.82 |
| *Yes* | 19/148 | 13.2 (8.3-20.5) | 0.93 (0.50-1.73) |  |
| Services don’t have medications I need |  |  |  |  |
| *No* | 55/473 | 11.9 (8.7-16.2) | 1 | 0.01 |
| *Yes* | 20/95 | 24.4 (15.9-35.6) | 1.99 (1.19-3.32) |  |
| Services frequently run out of medications |  |  |  |  |
| *No* | 56/487 | 11.9 (8.4-16.7) | 1 | 0.01 |
| *Yes* | 19/81 | 23.6 (16.3-33.0) | 2.05 (1.23-3.39) |  |
| Providers don’t understand my health problems |  |  |  |  |
| *No* | 60/433 | 14.7 (11.0-19.4) | 1 | 0.28 |
| *Yes* | 15/135 | 11.4 (7.3-17.3) | 0.77 (0.49-1.23) |  |

*Percentages and P-values are adjusted for the complex sampling strategy.*

**References**

Babor, T. F., J. C. Higgins-Biddle, J. B. Saunders, M. G. Monteiro and W. H. Organization (2001). AUDIT: The alcohol use disorders identification test: Guidelines for use in primary health care.

Boyd, J. E., E. P. Adler, P. G. Otilingam and T. Peters (2014). "Internalized Stigma of Mental Illness (ISMI) scale: a multinational review." Comprehensive Psychiatry **55**(1): 221-231.

Carey, K. B., M. P. Carey and P. S. Chandra (2003). "Psychometric evaluation of the alcohol use disorders identification test and short drug abuse screening test with psychiatric patients in India." The Journal of clinical psychiatry **64**(7): 767.

Chisholm, D., M. R. J. Knapp, H. C. Knudsen, F. Amaddeo, L. Gaite, B. Van Wijngaarden and E. S. Group (2000). "Client socio-demographic and service receipt inventory–European version: development of an instrument for international research: EPSILON Study 5." The British Journal of Psychiatry **177**(S39): s28-s33.

de Meneses-Gaya, C., A. W. Zuardi, S. R. Loureiro and J. A. S. Crippa (2009). "Alcohol Use Disorders Identification Test (AUDIT): An updated systematic review of psychometric properties." Psychology & Neuroscience **2**(1): 83.

Kessler, R. C. and T. B. Üstün (2004). "The world mental health (WMH) survey initiative version of the world health organization (WHO) composite international diagnostic interview (CIDI)." International journal of methods in psychiatric research **13**(2): 93-121.

Kowal, P., S. Chatterji, N. Naidoo, R. Biritwum, W. Fan, R. Lopez Ridaura, T. Maximova, P. Arokiasamy, N. Phaswana-Mafuya and S. Williams (2012). "Data resource profile: the World Health Organization Study on global AGEing and adult health (SAGE)." International journal of epidemiology **41**(6): 1639-1649.

Kroenke, K., R. L. Spitzer and J. B. Williams (2001). "The PHQ‐9: validity of a brief depression severity measure." Journal of general internal medicine **16**(9): 606-613.

Pal, H. R., R. Jena and D. Yadav (2004). "Validation of the Alcohol Use Disorders Identification Test (AUDIT) in urban community outreach and de-addiction center samples in north India." Journal of Studies on Alcohol **65**(6): 794-800.

Üstün, T. B., S. Chatterji, N. Kostanjsek, J. Rehm, C. Kennedy, J. Epping-Jordan and S. Saxena (2010). "Developing the World Health Organization Disability Assessment Schedule 2.0."
